# Cell-resolved spatial mapping of antibody-drug conjugate targets in breast cancer

**DOI:** 10.1016/j.tranon.2026.103068

**Published:** 2026-10-12

**Authors:** Yang Zhao, Zhixia Bai, Tao Li

**Affiliations:** a Department of Surgical Oncology, General Hospital of Ningxia Medical University, Yinchuan, Ningxia, China; b Department of Anesthesiology and Perioperative Medicine, General Hospital of Ningxia Medical University, Yinchuan, Ningxia, China; c Department of Gastrointestinal Surgery, People's Hospital of Ningxia Hui Autonomous Region, Yinchuan, Ningxia, China

**Keywords:** Antibody-drug conjugate, Breast cancer, Spatial transcriptomics, Single-cell analysis, HER2, TROP2, Tumor microenvironment

## Abstract

**Background:**

Antibody-drug conjugate (ADC) activity and target-dependent on-target/off-tumor toxicity depends in part on which cells express the target antigen, a feature that bulk assays cannot resolve.

**Methods:**

We analyzed a publicly available whole-transcriptome CosMx breast tumor dataset including 154,816 cells and 18,934 genes. Eleven clinically relevant ADC targets were profiled across seven annotated lineages. Findings were contextualized using TCGA-BRCA bulk transcriptomes (1,097 tumors and 114 normal tissues) and assessed in two public Xenium breast cancer specimens.

**Results:**

HER2 (*ERBB2*), TROP2 (*TACSTD2*), HER3 (*ERBB3*), *NECTIN4* and *MUC1* were enriched in tumor epithelium. B7-H3 (*CD276*) was highest in endothelial and myoepithelial cells, with similar mean expression in stromal and tumor cells. PSMA (*FOLH1*) was enriched in endothelium. TCGA-BRCA tumor-normal differences did not identify these cellular sources. No overall-survival association remained after multiple-testing correction or covariate adjustment. HER2 and TROP2 tumor enrichment recurred in both Xenium specimens, whereas *EGFR* was not tumor restricted.

**Conclusions:**

Cell-resolved spatial profiling separated ADC targets enriched in tumor epithelium from those expressed in vascular and stromal compartments. B7-H3 and PSMA localization was consistent with prior immunohistochemical reports and is confirmatory. These RNA-level results do not establish surface-protein abundance, ADC response, or clinical toxicity. Low-abundance microenvironmental findings remain based on a single discovery specimen and require protein-level validation. Protein-level confirmation, and correlation with ADC efficacy and toxicity, are required before these maps can inform clinical decisions.

## Highlights

• Spatial transcriptomics mapped 11 ADC targets across seven cell lineages.
• HER2 and TROP2 were consistently enriched in tumor epithelium.
• B7-H3 and PSMA transcripts localized mainly to non-tumor compartments.
• Bulk tumor-normal contrasts did not identify the expressing cell types.

## Graphical abstract

**Figure:**
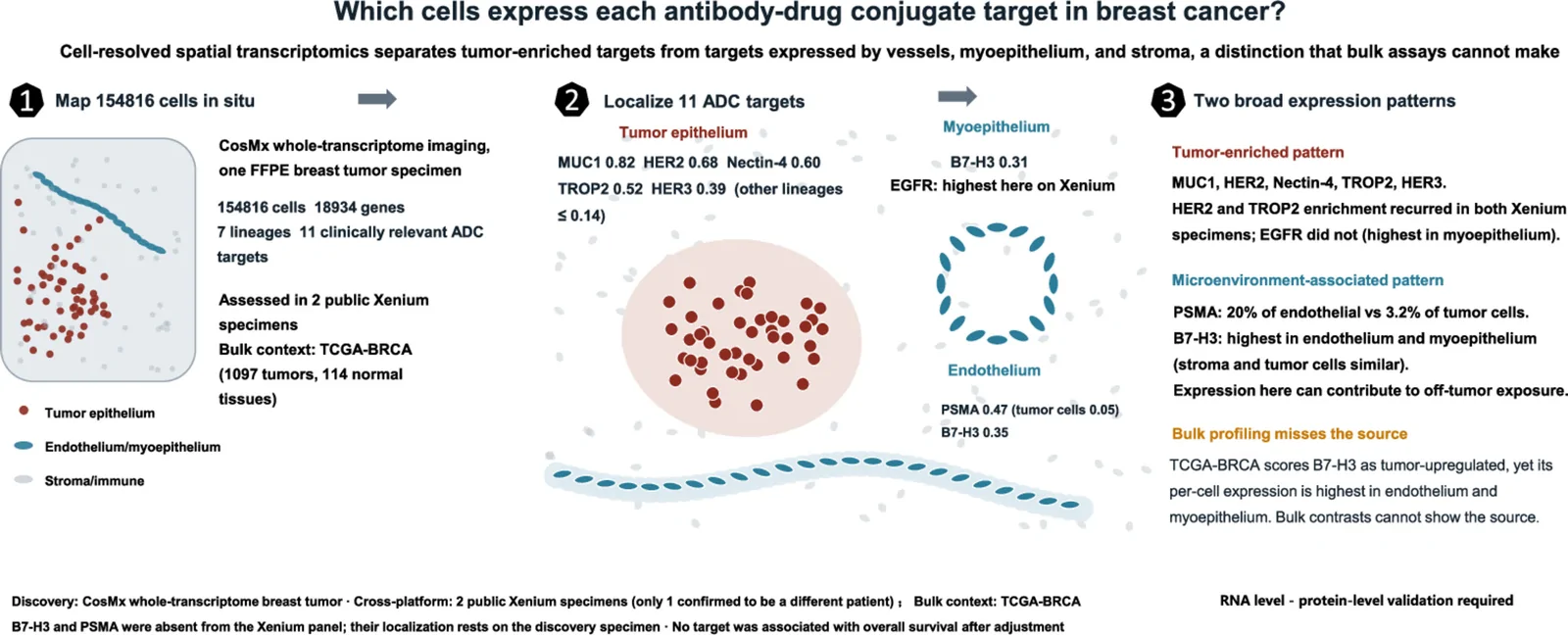

## Introduction

Breast cancer is the most frequently diagnosed cancer in women, with approximately 2.3 million new cases and 670,000 deaths worldwide in 2022 [1]. Antibody-drug conjugates (ADCs) link a target-specific antibody to a cytotoxic payload and are now central to breast-cancer treatment [2]. Trastuzumab emtansine established the class in HER2-positive disease [3], extending median progression-free survival from 6.4 to 9.6 months and median overall survival from 25.1 to 30.9 months relative to lapatinib plus capecitabine. Trastuzumab deruxtecan and the TROP2-directed agents Sacituzumab govitecan and datopotamab deruxtecan have since broadened their use across breast cancer subtypes [[4], [5], [6], [7]]. In the long-term DESTINY-Breast04 analysis, investigator-assessed median progression-free survival was 8.8 versus 4.2 months (hazard ratio, 0.36), and median overall survival was 22.9 versus 16.8 months (hazard ratio, 0.69), with trastuzumab deruxtecan versus treatment of the physician's choice in HER2-low advanced breast cancer [4]. In the final ASCENT analysis, Sacituzumab govitecan improved median progression-free survival to 4.8 versus 1.7 months (hazard ratio 0.41) and median overall survival to 11.8 versus 6.9 months (hazard ratio 0.51) in metastatic triple-negative breast cancer [5]. HER3, *NECTIN4*, folate receptor-α, B7-H3, *MUC1* and *CEACAM5* are among the additional targets in clinical development [[8], [9], [10]]; the molecular alterations of *MUC1* and their therapeutic exploitation have been reviewed recently [11].

For an ADC, tissue-level abundance is not enough. The target must also be located on the relevant cells. Expression in stroma, vasculature or normal epithelium can contribute to target-dependent on-target/off-tumor toxicity, but ADC toxicity is multifactorial and also reflects payload cytotoxicity, platform-related properties, and host factors [12]. The clinical burden can nonetheless be substantial. In the updated DESTINY-Breast04 analysis, adjudicated drug-related interstitial lung disease or pneumonitis occurred in 12.1% of patients receiving trastuzumab deruxtecan, including 1.1% grade 5 events [4]. In the final ASCENT analysis, the most common grade 3 or higher treatment-emergent adverse events with Sacituzumab govitecan included neutropenia (52%) and diarrhea (12%) [5]. Conversely, target-negative tumor cells may escape direct binding, although a membrane-permeable payload can affect neighboring cells through a bystander effect [13]. Bulk assays and tissue-averaged immunohistochemistry do not resolve the cellular source of a target signal. The broader importance of tissue architecture extends beyond ADCs to other tumor-directed delivery platforms, including nanocarriers influenced by vascular permeability and retention, as well as stimuli-responsive nanoparticles under development for breast cancer [14,15].

Cell-resolved target assessment is already used in the chimeric antigen receptor T-cell field to anticipate on-target/off-tumor effects [16]. For ADCs, bulk surveys have catalogued target expression across tumors and normal tissues, including a 54-target pan-cancer analysis [17]. A post-mortem breast cancer series also compared metastases with matched normal organs [18]. In high-grade serous ovarian cancer, dissociated single-cell RNA sequencing identified clinical ADC targets that were most often restricted to cancer cells [19]. Spatial profiling has therefore been proposed as a means of interpreting target availability in tissue context [20]. Breast single-cell atlases have examined heterogeneity in ERBB2 and TACSTD2, but primarily within the malignant epithelial compartment [21].

Few studies have spatially identified multiple ADC targets across malignant and non-malignant compartments in breast cancer. Imaging-based transcriptomics can provide this information while preserving histological structure [[22], [23], [24], [25]]. We therefore mapped 11 ADC targets in a publicly available whole-transcriptome breast cancer specimen, examined the recurrence of selected patterns in two public Xenium specimens, and compared the findings with TCGA-BRCA bulk transcriptomes. The study is descriptive. Its purpose is to identify which cells contribute to each target transcript and to show where tissue-level abundance may obscure that source.

## Materials and methods

### Single-cell spatial dataset

We used the public CosMx Spatial Molecular Imager Human Whole Transcriptome FFPE breast dataset (Bruker Spatial Biology, "breast discovery" release, sample S0) [22]. The dataset contains one tumor specimen, which served as the discovery sample. Selected findings were examined in two additional public Xenium specimens (the cross-specimen Xenium analysis). We obtained the cell-by-gene matrix, cell metadata, and cell polygons from the public repository. The complete dataset contained approximately 645,000 cells. To make the analysis tractable using standard hardware, we drew a reproducible random subsample (seed = 1) and retained cells with at least 50 detected RNA molecules. This yielded 154,816 cells. Removing negative-control and system-control probes left 18,934 genes. Global coordinates were converted from pixels to micrometers using a scale of 0.12028 µm per pixel. We also split the retained cells into two random halves and compared cell-type mean target-expression matrices to assess sensitivity to subsampling.

### Clustering, annotation and differential expression

Analyses were performed in R 4.5.2 with Seurat 5.5.1 [26]. Counts were log-normalized to counts per 10,000, then subjected to a natural-log transformation. We scaled the 2,000 most variable genes and used 30 principal components to construct a shared-nearest-neighbor graph. Louvain clustering was run at resolution 0.5, followed by UMAP embedding. Clusters were assigned to seven lineages using module scores for canonical markers and established breast cell-type signatures [27]. The lineages were tumor epithelial, fibroblast/stromal, myeloid, B/plasma, T, myoepithelial, and endothelial. One-versus-rest Wilcoxon rank-sum tests identified positive cell-type markers, with at least 10% detection, a minimum log2 fold-change of 0.25, and at most 500 cells per group. P values were adjusted by the Benjamini-Hochberg method. These cell-level tests treat cells from one specimen as independent observations. We therefore used them to describe within-sample marker specificity and placed greater weight on effect sizes than on nominal P values.

### ADC-target analysis

We examined eleven clinically relevant ADC targets: *ERBB2* (HER2), *TACSTD2* (TROP2), *ERBB3* (HER3), *NECTIN4, FOLR1* (FRα), *CEACAM5* (CEA), *CD276* (B7-H3), *MUC1, MET, EGFR* and *FOLH1* (PSMA). For each cell type, we calculated mean log-normalized expression and the number and fraction of cells with detectable expression. Per-cell expression was also mapped in tissue coordinates.

### Cross-platform single-target detection comparison

To describe cross-platform differences in single-target detection, we compared *ERBB2* and *TACSTD2* between the whole-transcriptome CosMx dataset and a 313-gene Xenium breast panel [23]. For each dataset, we calculated the fraction of tumor cells with zero counts for the target gene. The assays were applied to different specimens and used different chemistries and panel sizes; this descriptive comparison was not designed to isolate a platform or panel-design effect.

### Receptor subtype, spillover control and spatial organization

For each target, we report the number and fraction of expressing cells, along with the mean expression. This provides cellular support for findings in low-abundance microenvironments. To assess whether *FOLH1* and *CD276* enrichment could reflect transcript spillover across cell boundaries, we built a k-nearest-neighbor graph (k = 10) from cell centroids. We calculated the proportion of tumor-epithelial neighbors for each cell, then compared expression in the enriched lineage between cells with few (<25%) and many (>50%) tumor neighbors. The receptor phenotype was inferred from tumor-cell expression of *ESR1, PGR,* and *ERBB2*, with *GATA3* and *MKI67* as context. This RNA-based classification is not equivalent to immunohistochemical receptor status. Cell-type neighborhood enrichment followed the approach implemented in Squidpy [28]. For each ordered cell-type pair, we compared the observed number of graph edges with a null distribution from 100 permutations of cell-type labels and expressed the result as a z-score.

### Bulk transcriptomic contextualization (TCGA-BRCA)

TCGA-BRCA gene-level expression (log2(normalized count + 1)) and clinical data were obtained from the UCSC Xena TCGA hub [29,30]. Sample barcodes identified 1,097 primary tumors and 114 solid-tissue normals. In this dataset, *NECTIN4* is recorded under its alias *PVRL4* and was mapped accordingly. Tumor-versus-normal differences were tested for each target by the Wilcoxon rank-sum test with Benjamini-Hochberg adjustment. Overall survival was derived from vital status and time to death or last follow-up. The full survival cohort contained 1,080 patients. For each target, we fitted a univariable Cox model using continuous expression and performed a median-split log-rank test. We interpreted these P values in the context of the 11 tested targets and did not search for additional endpoints and subgroups. Multivariable Cox models were fitted separately in the 808 patients with complete data for age, pathologic stage, and PAM50 intrinsic subtype.

### Cross-specimen validation on Xenium

We analyzed two additional public Xenium breast cancer specimens: the Human Breast invasive ductal carcinoma sample and the breast cancer reference block [23]. The invasive ductal carcinoma specimen came from a different patient. Public metadata did not establish patient-level independence of the reference block. We used the vendor-supplied graph-based clusters and assigned cells to the same lineages by marker scores. Four ADC targets were represented in the Xenium panel: *ERBB2, TACSTD2, MUC1,* and *EGFR*. For each target, we compared detection fractions between tumor epithelial and non-tumor cells. Detection fraction was chosen because absolute expression is not directly comparable across platforms with different panel sizes and sequencing depths.

## Results

### A seven-lineage single-cell spatial map of the breast tumor

Unsupervised clustering produced 15 clusters, which were assigned to seven cell types (Fig. 1). Tumor epithelium was the largest compartment (95,803 cells; 61.9%). Fibroblast/stromal and myeloid cells each accounted for 10.3%, followed by B/plasma cells (6.9%), T cells (5.3%), myoepithelial cells (2.7%), and endothelial cells (2.6%). Quality-control distributions are shown in Supplementary Fig. S2. These populations occupied coherent tissue regions, with tumor epithelium forming contiguous fields and glandular structures (Fig. 1B). One-versus-rest testing identified 4,449 positive markers. The leading markers matched the assigned lineages, including *PIP, SERHL2,* and *MLPH* in the tumor epithelium; *COL1A1, LUM,* and *POSTN* in the stroma; and *EGFL7, PLVAP,* and *FLT1* in the endothelium (Fig. 2; Supplementary Table S4). The same correspondence was seen for myeloid (*APOE, LYZ, C1QC*), B/plasma (*IGKC, IGHA1, JCHAIN*), T (*GNLY, GZMA, ITGAE*) and myoepithelial cells (*ACTA2, MCAM, CSPG4*).

**Fig. 1:**
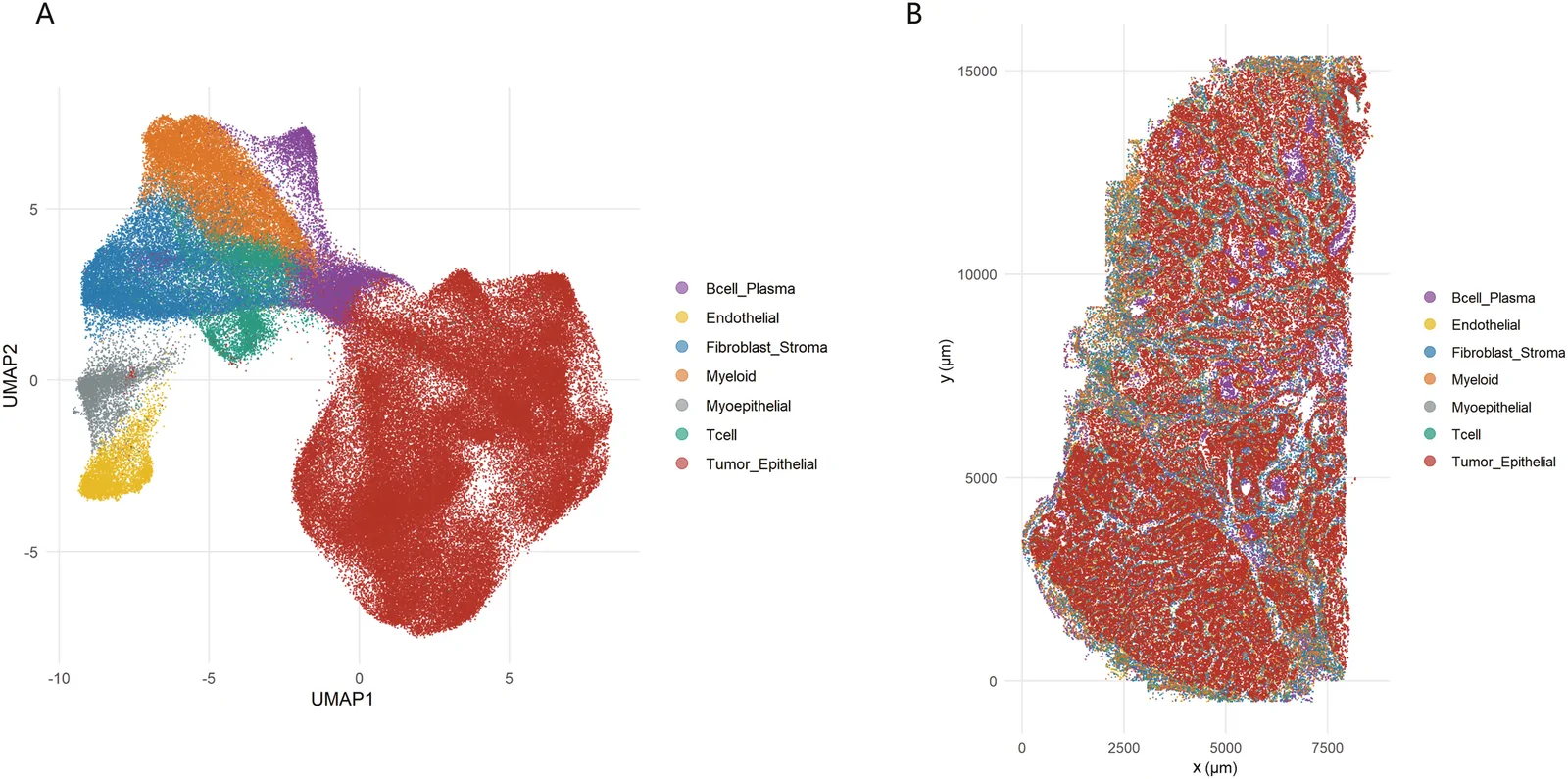
Single-cell spatial map of the breast tumor. (A) Uniform manifold approximation and projection (UMAP) of 154,816 analyzed cells from one CosMx breast tumor specimen, colored by seven annotated cell types. (B) Spatial distribution of the same cells using tissue coordinates in micrometers. Each point represents a cell centroid. The same cell-type color key is used in both panels.

**Fig. 2:**
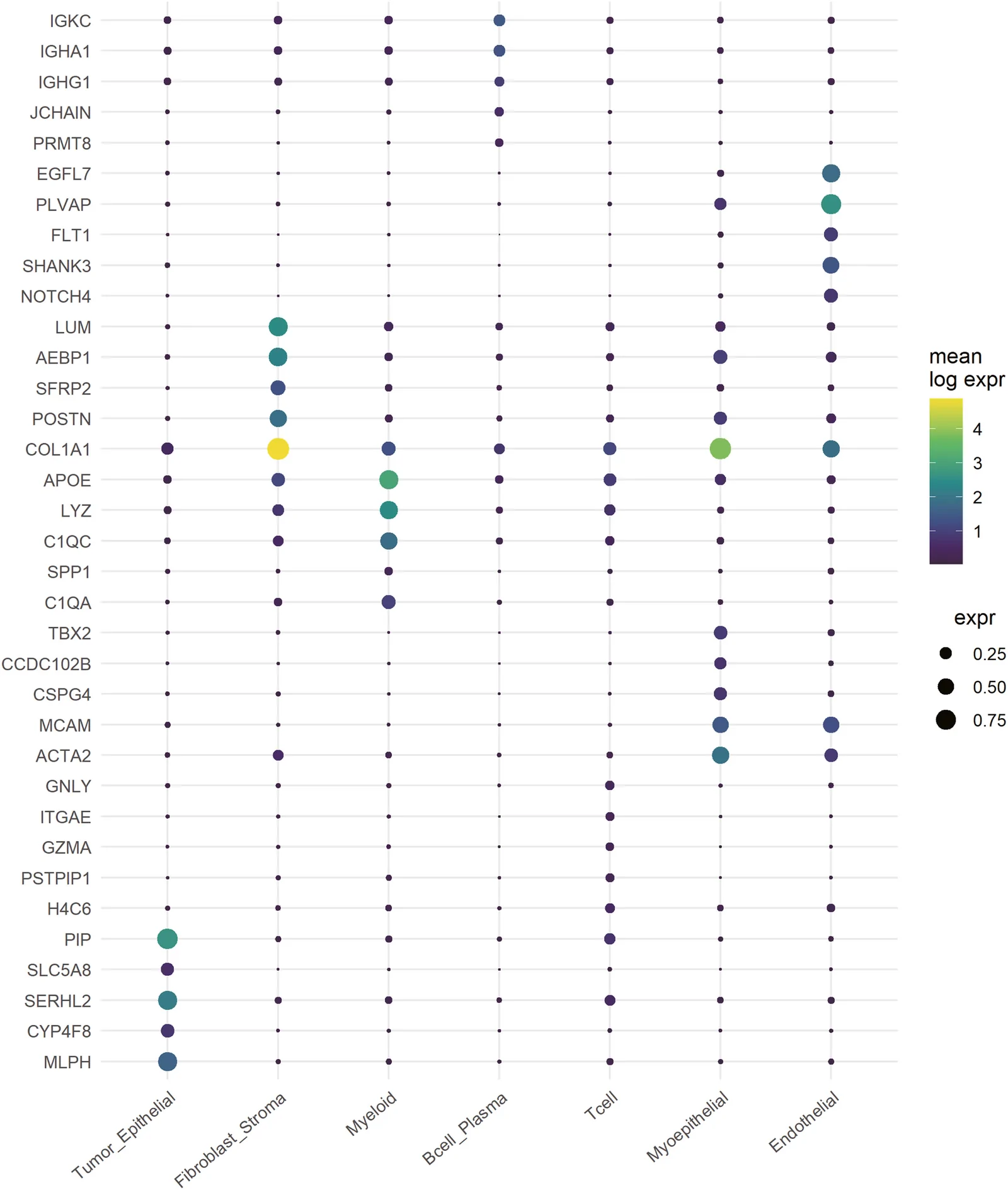
Cell-type marker expression in the discovery specimen. The five positive one-versus-rest markers with the largest average log2 fold change are shown for each of seven cell types. Dot color represents mean log-normalized expression across all cells in the indicated lineage, including zero-count cells. Dot size represents the fraction of cells with a nonzero gene count. The size-key values are fractions: 0.25, 0.50 and 0.75 correspond to 25%, 50% and 75%, respectively. Expression was normalized to 10,000 counts per cell and transformed using ln(1 + x). Marker-test results are provided in Supplementary Table S4.

### ADC targets show tumor-enriched and microenvironment-enriched patterns

The eleven ADC targets followed two broad patterns (Fig. 3). *MUC1* (mean log-normalized expression 0.82), HER2/*ERBB2* (0.68), *NECTIN4* (0.60), TROP2/*TACSTD2* (0.52) and HER3/*ERBB3* (0.39) were highest in tumor epithelium. Their mean expression in non-malignant compartments was 0.14 or lower. Spatial maps showed that HER2 and TROP2 expression were associated with the tumor-epithelial regions of the section (Fig. 4).

**Fig. 3:**
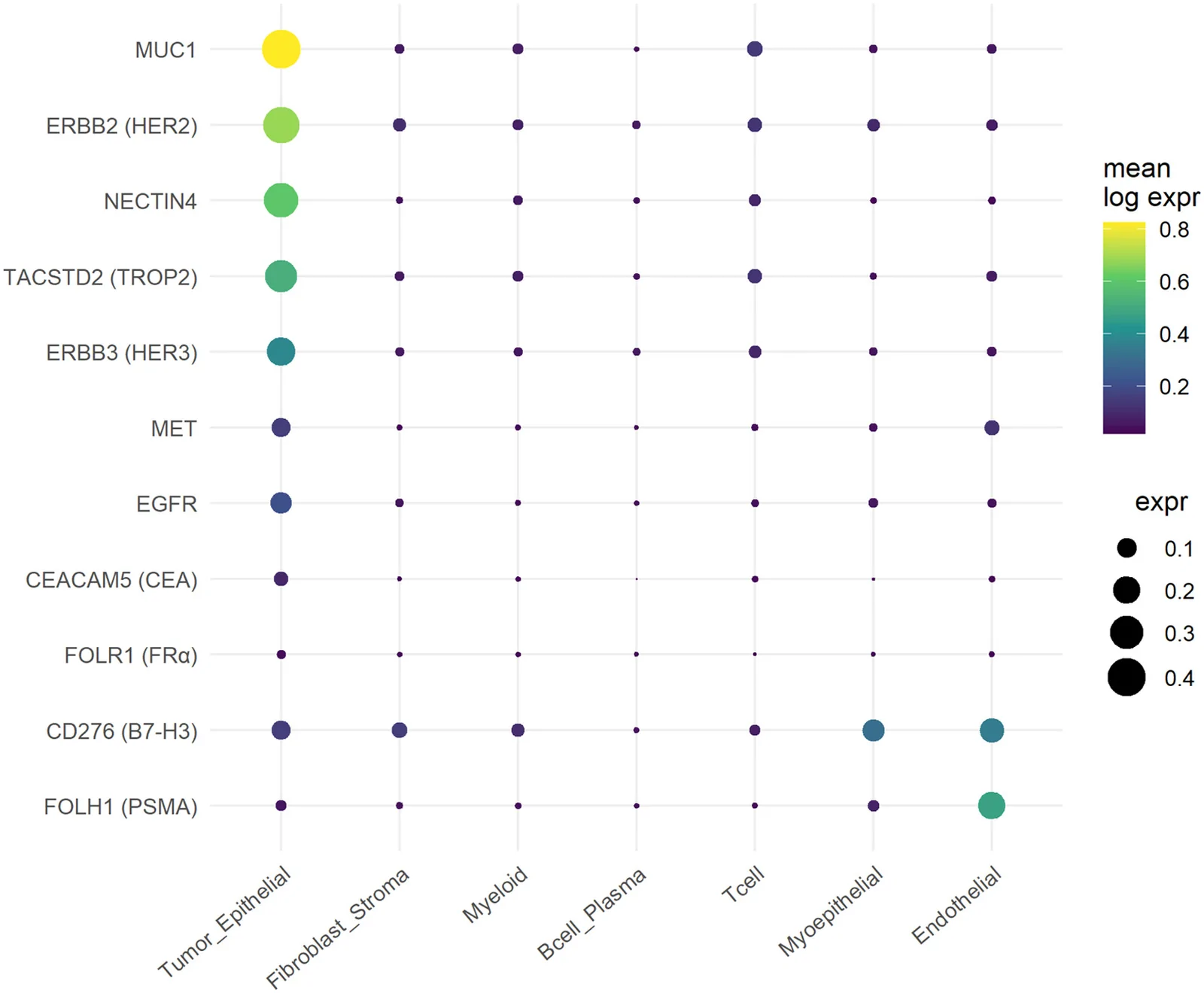
ADC-target expression across cell types. Dot plot of 11 antibody-drug conjugate (ADC) targets in the CosMx discovery specimen. Dot color represents mean log-normalized expression across all cells in each lineage, including zero-count cells. Dot size represents the fraction of cells with a nonzero gene count; size-key values of 0.1-0.4 correspond to 10%-40%. Expression was normalized to 10,000 counts per cell and transformed using ln(1 + x). MUC1, HER2, NECTIN4, TROP2 and HER3 had their highest mean expression in tumor epithelium. B7-H3 was highest in endothelial and myoepithelial cells, and PSMA was highest in endothelium. Cell counts and detection fractions are provided in Supplementary Table S1. These measurements concern RNA expression.

**Fig. 4:**
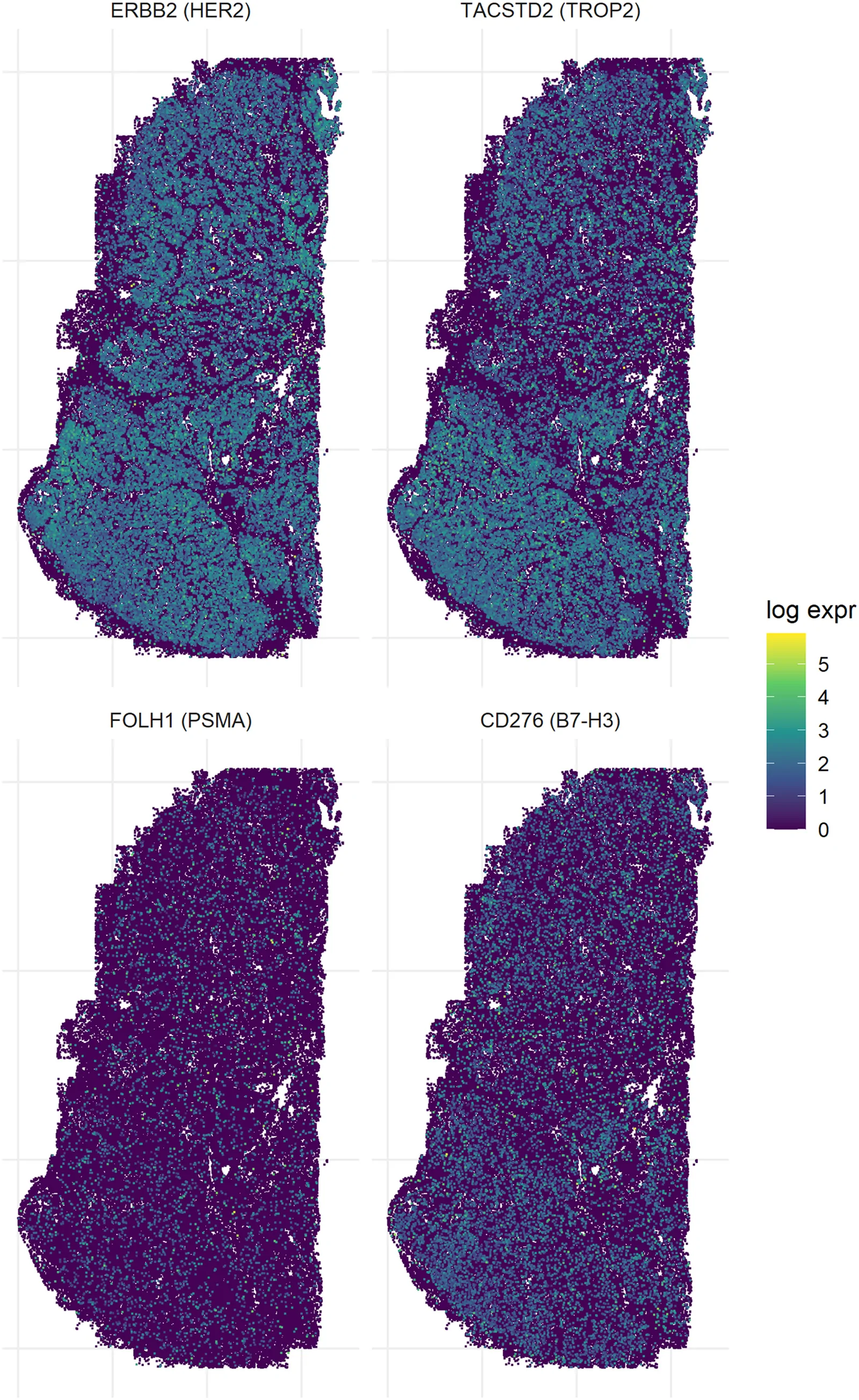
Spatial expression of representative ADC targets. Per-cell expression of HER2 (ERBB2, upper left), TROP2 (TACSTD2, upper right), PSMA (FOLH1, lower left) and B7-H3 (CD276, lower right) in the CosMx discovery specimen. Each point represents a cell centroid. The four panels use the same tissue coordinates and a common expression color scale. Expression was normalized to 10,000 counts per cell and transformed using ln(1 + x). The maps show transcript localization and do not establish surface-protein abundance.

Other targets were expressed preferentially outside the tumor compartment. B7-H3/*CD276* was highest in endothelial (0.35) and myoepithelial cells (0.31), followed by fibroblast/stromal and tumor cells (both 0.16). PSMA/*FOLH1* was concentrated in endothelium (0.47), whereas mean tumor-cell expression was 0.05. This distribution agrees with the reported localization of PSMA to tumor-associated neovasculature rather than breast carcinoma cells [31]. *FOLR1* and *CEACAM5* remained low across all cell types in this specimen (≤0.10).

The microenvironmental findings were supported by the number of cells expressing (Supplementary Table S1). PSMA was detected in 20.0% of endothelial cells (817 of 4,076) and 3.2% of tumor cells. B7-H3 was detected in 16.0% of endothelial cells, 12.9% of myoepithelial cells, and 9.5% of tumor cells. Low PSMA expression in tumor cells argues against tumor-derived transcript spillover as the main explanation for endothelial enrichment. In the neighborhood analysis, endothelial cells with fewer than 25% tumor neighbors still had a mean PSMA expression of 0.39 (n = 2,282), compared with 0.05 in tumor cells. B7-H3 enrichment in stroma, endothelium and myoepithelium was also retained in cells located away from tumor epithelium (Supplementary Table S2).

### Single-target detection differed between platforms and specimens

In the CosMx whole-transcriptome specimen, 60% of tumor cells had zero *ERBB2* counts, and 68% had zero *TACSTD2* counts. In the targeted 313-gene Xenium specimen, the corresponding fractions were 3.5% and 11%. Because the assays were applied to different tissue specimens and used different chemistries and panel sizes, these differences cannot be attributed solely to assay design. They nevertheless caution against interpreting a zero count as absence of target expression over spatial transcriptomic platforms.

### Tumor receptor phenotype and spatial organization

Among tumor cells, *ERBB2* was the most highly expressed receptor gene (mean 0.68; detected in 36.6% of cells). *ESR1* (0.09; 5.8%) and *PGR* (0.04; 2.7%) were low. This RNA profile is compatible with, but not diagnostic of, a HER2-enriched or hormone-receptor-negative phenotype. The HER2 and TROP2 findings should therefore be interpreted in the context of these receptors.

Neighborhood analysis showed strong self-association of the tumor epithelium (z = 507) and negative associations with all non-malignant cell types. The largest negative values were observed in myeloid cells (z = -308) and fibroblast/stromal cells (z = -294). Among non-malignant populations, fibroblasts co-localized most strongly with myeloid cells (z = 131), whereas endothelial cells co-localized most strongly with myoepithelial cells (z = 137; Supplementary Fig. S3). Subsampling had little effect. Two random halves of the retained cells produced nearly identical cell-type mean target-expression matrices (Pearson r = 0.998).

### HER2 and TROP2 tumor enrichment recurs in additional Xenium specimens

Both Xenium specimens were public datasets distinct from the CosMx discovery sample, although only the invasive ductal carcinoma specimen was known to come from a different patient. We therefore assessed cross-specimen recurrence without treating the reference block as a separate patient-level replicate (Supplementary Fig. S4, Supplementary Table S3). Because panel content and sequencing depth differed, the comparison used detection fractions. Ranking cell types by mean expression gave the same ordering. Tumor epithelium had the highest HER2/*ERBB2* and TROP2/*TACSTD2* expression in all six specimen-by-target comparisons. In the invasive ductal carcinoma specimen, for example, TROP2 was detected in 84% of tumor cells and 26% of non-tumor cells. *MUC1* showed the same pattern where it was included in the panel. *EGFR* differed. In both Xenium specimens, *EGFR* was detected as often as or more often in non-tumor cells, with the highest levels in myoepithelial cells.

### Bulk tumor-versus-normal expression

In TCGA-BRCA (1,097 tumors and 114 normal tissues), six targets were higher in tumor tissue: *CEACAM5* (log2 fold-change +4.1), *MUC1* (+2.1), *CD276* (+0.85), *ERBB3* (+0.85), *NECTIN4* (+0.75) and *ERBB2* (+0.54). All adjusted P values were below 0.001 (Fig. 5; Supplementary Table S5). *TACSTD2* did not differ between groups (log2 fold-change -0.29; P = 0.06). Four targets were lower in tumors: *FOLR1* (-3.8), *EGFR* (-3.5), *MET* (-2.4) and *FOLH1* (-1.6). These bulk contrasts are influenced by tissue composition and do not identify the cell type expressing the gene. B7-H3 illustrates the distinction, as it was higher in bulk tumor tissue but arose mainly from endothelial, myoepithelial and stromal cells in the spatial data.

**Fig. 5:**
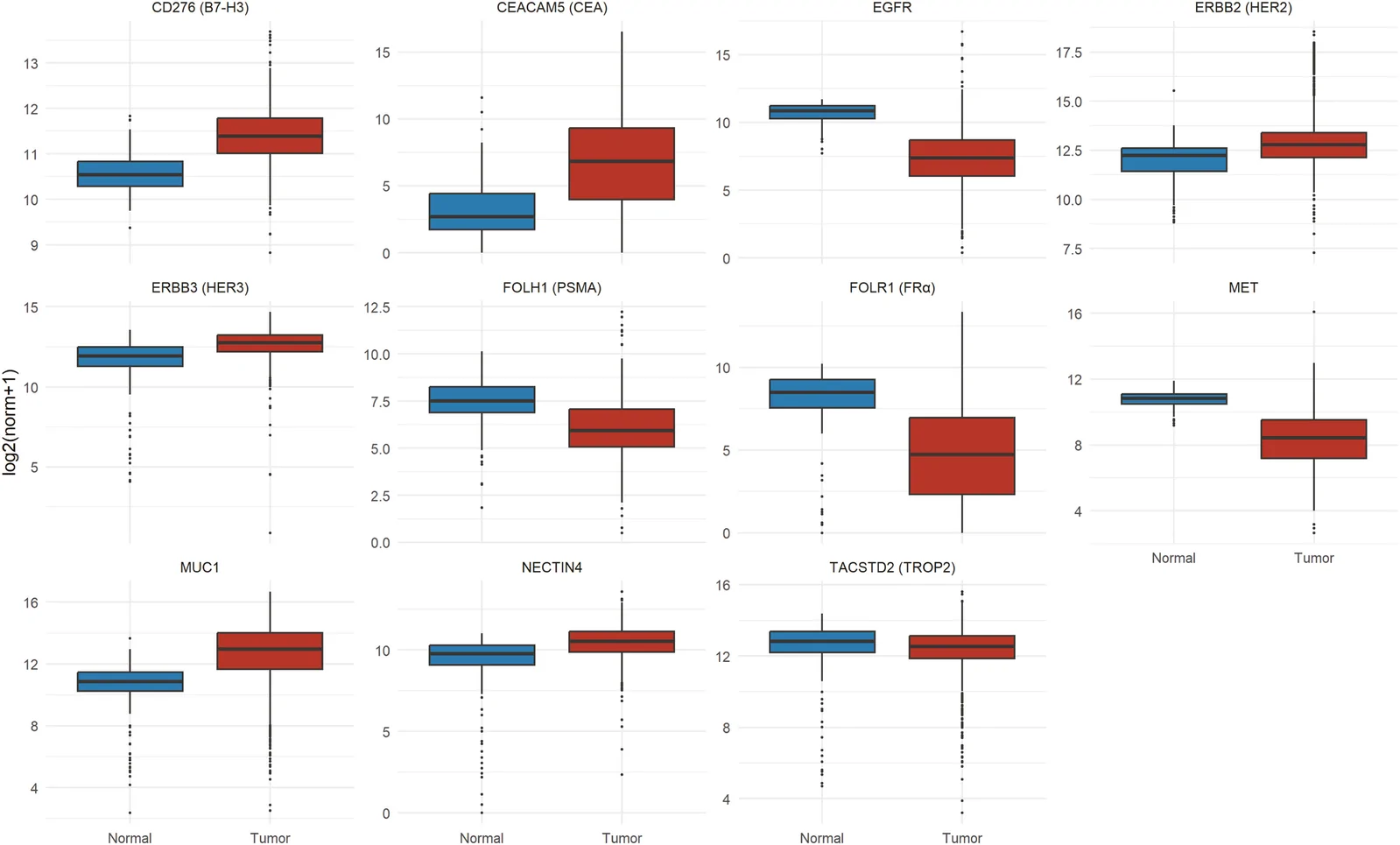
ADC-target expression in TCGA-BRCA tumors and normal tissues. Box plots show log2(normalized count + 1) expression of 11 ADC-target genes in primary tumors (n = 1,097; red) and solid-tissue normal samples (n = 114; blue). The center line is the median, boxes span the 25th-75th percentiles, and whiskers extend to the most extreme values within 1.5 times the interquartile range. Points beyond the whiskers are outliers. The y-axis range differs between gene panels. Two-sided Wilcoxon comparisons and Benjamini-Hochberg-adjusted P values are reported in Supplementary Table S5.

### No ADC target shows a strong association with overall survival

Among the 1,080 patients with survival data, no univariable association remained significant after correction for eleven targets. The lowest nominal P values in this full cohort were observed for *NECTIN4* in the continuous Cox model (P = 0.040) and the median-split log-rank test (P = 0.053; Supplementary Fig. S1; Supplementary Table S6). The complete-covariate subset contained 808 patients. In this subset, *CEACAM5* had a nominal univariable association (P = 0.019), which weakened after adjustment for age, pathologic stage, and PAM50 subtype (hazard ratio 1.22; P = 0.071; Benjamini-Hochberg-adjusted P = 0.478). No target was associated with overall survival after covariate adjustment (Supplementary Table S7). These analyses do not support a strong single-gene prognostic effect, although the unselected cohort and limited event count constrain the inference.

## Discussion

At the RNA level, the eleven ADC targets separated into two expression patterns in the discovery specimen. HER2, TROP2, HER3, *NECTIN4* and *MUC1* were concentrated in tumor epithelium. Among these, *MUC1* had the highest mean expression, consistent with its long-standing status as a tumor-associated antigen and with continuing efforts to exploit *MUC1* alterations therapeutically [11]. B7-H3 and PSMA were more prominent in stromal or vascular compartments. Bulk and tissue-averaged assays cannot resolve this difference in cellular source. Recovery of the established PSMA neovascular pattern provides an internal check on the cell assignments. These data do not estimate the therapeutic index, which also depends on surface protein abundance, internalization, expression in vital normal tissues, and payload and linker chemistry.

Localization alone does not indicate whether a compartment contains sufficient antigen to affect treatment. PSMA is a useful caution. We detected *FOLH1* in 20% of endothelial cells, but quantitative immunohistochemistry found PSMA staining in only a small minority of breast-cancer neovessels [32]. Those authors questioned the viability of PSMA-directed theranostics in this disease. A cell type can therefore be the dominant source of a low-abundance transcript without presenting a clinically meaningful antigen burden. Our study locates the RNA signal but does not quantify surface protein or predict efficacy or toxicity.

The bulk and spatial examinations answered different questions. TCGA-BRCA identified targets whose total expression differed between tumor and normal tissue. It could not show whether the signal came from malignant cells. B7-H3 was higher in bulk tumor tissue but mapped mainly to endothelium, myoepithelium, and stroma in the spatial specimen. This is not a contradiction. Tumor-versus-normal contrasts are influenced by tissue composition, so we do not interpret bulk upregulation as evidence of tumor-cell specificity.

Prior work defines the limits of the study's novelty. Single-cell surveys have already been used to identify non-malignant expression of CAR-T targets [16]. ADC-target catalogues are available for pan-cancer and breast cancer tissues [17,18], and dissociated single-cell data have been used to assess ADC target restriction in ovarian cancer [19]. B7-H3 expression in tumor endothelium and fibroblasts has been reported [33], as has PSMA expression in breast cancer neovasculature [31,32]. Our contribution is narrower. We assign 11 ADC-target transcripts to cell types in intact breast tissue, including the myoepithelial compartment, and assess selected targets in 2 additional specimens using another platform. The recovery of the known B7-H3 and PSMA localizations supports the approach but does not constitute a new biological discovery.

Tumor epithelium occupied spatially segregated regions, apart from stromal, immune, and vascular populations (Supplementary Fig. S3). Together with the neighborhood control, this pattern makes simple transcript spillover an unlikely explanation for the B7-H3 and PSMA findings. The discovery tumor had low *ESR1* and *PGR* expression and comparatively high *ERBB2*. HER2 and TROP2 localization should therefore be interpreted within the context of their respective receptors rather than generalized across all breast cancer subtypes.

Cross-specimen support was strongest for HER2 and TROP2. Their enrichment in tumor epithelium recurred in both Xenium specimens and on a second imaging platform. The invasive ductal carcinoma specimen came from a different patient, but the public metadata did not establish patient-level independence for the reference block. The evidence therefore represents recurrence across three specimens and two platforms, not three independent patients. *EGFR* was highest in myoepithelial cells in both Xenium specimens, showing that tumor restriction cannot be assumed for every target. B7-H3 and PSMA were absent from the targeted panel, so their localization remains based on the discovery specimen.

The survival analyses provide no evidence that any target is an independent prognostic marker. Nominal associations differed between the full cohort and the complete-covariate subset and did not survive correction for or adjustment for covariates. This null result should not be read as proof that the target expression has no prognostic relevance in any subgroup. The cohort was unselected, the number of events was limited, and we deliberately avoided exploratory analyses by subtype or alternative endpoints.

Translating these transcript-level maps into clinical decisions will require protein-level measurement and direct correlation with ADC efficacy and toxicity. We regard this as the central next step, rather than a peripheral caveat. A practical sequence would begin with multiplexed immunofluorescence or imaging mass cytometry on matched tissue sections, using the lineage markers applied here to reproduce the cell assignments alongside antibodies directed against the ectodomains engaged by ADCs. Simultaneous RNA and protein co-detection on the same section would then allow the transcript-to-protein relationship to be assessed cell-by-cell rather than assumed. Compartment-resolved quantitative scoring, as applied to PSMA in breast-cancer neovasculature [32], could subsequently convert localization into an estimate of antigen burden within each compartment. Clinical correlation would require complementary study designs. For efficacy, pretreatment tumor blocks from patients subsequently treated with a defined ADC could be scored separately for tumor-cell and microenvironmental target protein expression and related to objective response and progression-free survival. Assessment of toxicity would require additional evidence from relevant normal tissues and organ-specific adverse events in ADC-treated cohorts, because protein expression within the tumor microenvironment alone cannot establish on-target/off-tumor toxicity. Only outcome-linked studies of this kind can determine whether compartment-specific expression patterns such as those reported here for B7-H3 and PSMA have measurable clinical consequences. The spatial framework may also be relevant beyond ADCs. Nanoparticle and other tumor-directed delivery systems are influenced by vascular permeability, stromal barriers, and the organization of the tumor microenvironment [14,15]. Although these mechanisms differ from antigen-dependent ADC targeting, they reinforce the broader value of resolving delivery-relevant compartments within intact tissue. Until protein-level and outcome-linked data become available, however, the present findings should be interpreted as a hypothesis-generating guide to where such validation should be directed, not as evidence of ADC efficacy or safety.

## Limitations

The whole-transcriptome discovery analysis is based on one tumor. HER2 and TROP2 were examined in two additional public Xenium specimens, but only one was clearly documented as a different patient. B7-H3 and PSMA were not present in the targeted validation panel. Their low-abundance stromal and vascular patterns, therefore, require confirmation in larger, patient-level cohorts across breast cancer subtypes. Cell annotations were marker-based rather than reviewed against matched histopathology. Differential expression tests also treated cells from a single specimen as independent units, which can inflate statistical confidence. Effect sizes and marker identity are more informative here than nominal cell-level P values. Public metadata did not consistently provide specimen-level sex and gender information, and these variables were not analyzed.

Whole-transcriptome imaging was sparse for individual genes, limiting per-cell quantification. The bulk and survival analyses were based on a single cohort, and no signal of survival remained after adjustment. Most importantly, every target measurement was messenger RNA. ADC binding depends on the availability of an accessible cell-surface protein, whose RNA abundance cannot be measured directly. We did not assess protein localization, internalization, drug response, or toxicity. The findings are therefore descriptive and hypothesis-generating.

## Conclusion

Single-cell spatial transcriptomics separated ADC targets enriched in tumor epithelium from targets expressed in stromal and vascular cells. HER2 and TROP2 showed the most consistent tumor enrichment across the three profiled specimens, whereas *EGFR* did not. These RNA-level findings should inform the design of protein-level, multi-patient validation studies. They cannot substitute for direct measurement of surface antigen or ADC activity. Compartment-resolved protein confirmation, followed by correlation with ADC response and toxicity in treated cohorts, is the necessary next step before any of these observations is applied clinically.

## Ethics approval and informed consent

This study used only publicly available, de-identified datasets and involved no direct participant contact or new data collection. Ethical approval and informed-consent procedures for the source studies were reported in the original dataset publications. No additional ethics approval or informed consent was required for this secondary analysis.

## Funding

This work was supported by the Ningxia Natural Science Foundation [grant numbers 2026AAC031176, 2026AAC031199 and 2025AAC030748], the National Natural Science Foundation of China [grant number 82260716], and the Regional Key Research and Development Program of the Ningxia Hui Autonomous Region [grant number 2026BEG02001]. The funders had no role in study design, data collection, analysis, interpretation, manuscript preparation, or the decision to submit the article for publication.

## Declaration of competing interest

The authors declare that they have no known competing financial interests or personal relationships that could have appeared to influence the work reported in this article.

## Data and code availability

All analyzed datasets are publicly available. The discovery dataset was the CosMx SMI Human Whole Transcriptome FFPE breast dataset (breast discovery release, sample S0; Bruker Spatial Biology; https://nanostring.com/products/cosmx-spatial-molecular-imager/ffpe-dataset/). The validation datasets were the Xenium Human Breast invasive ductal carcinoma specimen (Xenium_V1_FFPE_Human_Breast_IDC_With_Addon) and the Human Breast Cancer reference block (Xenium_FFPE_Human_Breast_Cancer_Rep1) from the 10x Genomics Datasets portal (https://www.10xgenomics.com/datasets). TCGA-BRCA gene expression and clinical data were obtained from the UCSC Xena TCGA hub (https://tcga.xenahubs.net). Analysis scripts and package versions are provided in the Supplementary_Code.zip file. Supplementary Tables S1-S7 and Fig. source data are provided as machine-readable Excel workbooks.

## Declaration of generative AI and AI-assisted technologies in the manuscript preparation process

During the preparation of this work, the authors used OpenAI ChatGPT and Codex to assist with code development and review, language editing, and preparation of submission files. After using these tools, the authors reviewed and edited the content as needed and take full responsibility for the published article.

## CRediT authorship contribution statement

**Yang Zhao:** Writing – original draft, Software, Resources, Project administration, Methodology, Investigation. **Zhixia Bai:** Writing – original draft, Visualization, Validation, Supervision, Software, Funding acquisition, Formal analysis, Data curation. **Tao Li:** Writing – review & editing, Supervision, Project administration, Investigation, Funding acquisition, Conceptualization.

## Declaration of competing interest

The authors declare that they have no known competing financial interests or personal relationships that could have appeared to influence the work reported in this paper.
