# Supplementary material for: Cell-resolved spatial mapping of antibody-drug conjugate targets in breast cancer
Source: Transl Oncol. 2026 Oct 12;74:103068. doi: 10.1016/j.tranon.2026.103068 (PMC13679307; doi:10.1016/j.tranon.2026.103068)
Supplement: Supplementary file 1 [file mmc1.pdf]

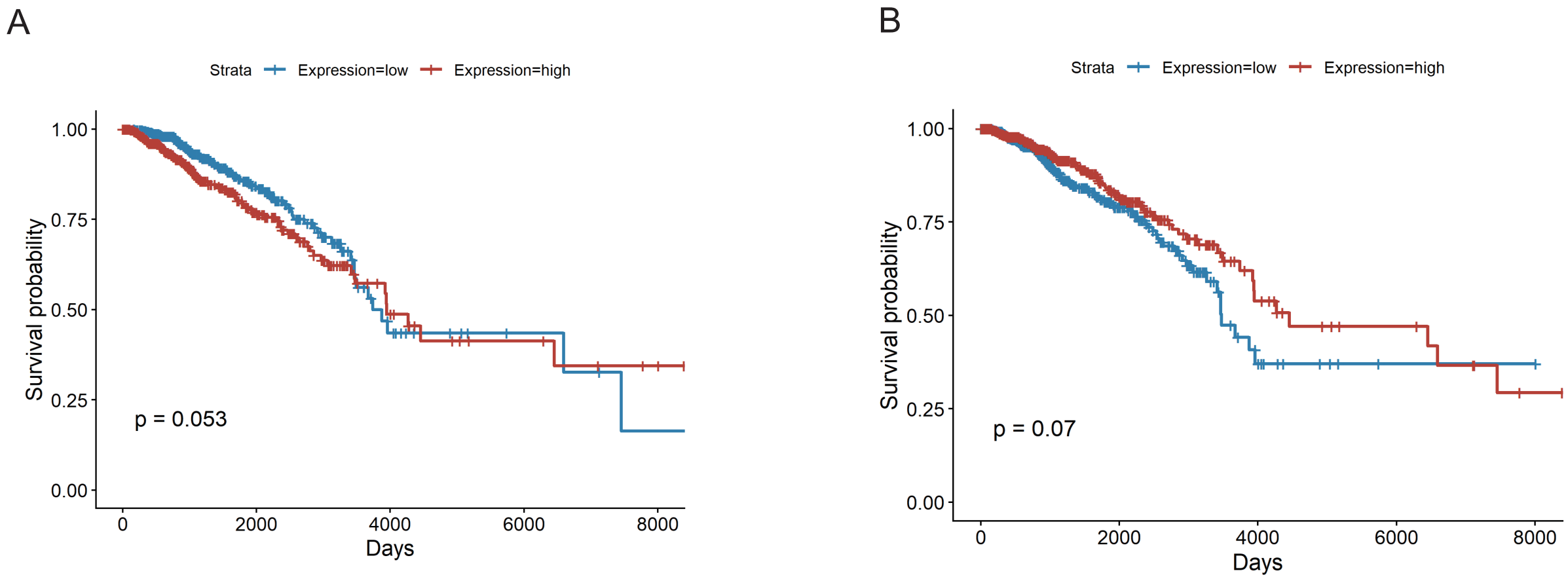

Figure S1. Overall survival by median-split target expression. Kaplan-Meier curves for (A) NECTIN4 and (B) TACSTD2 in 1,080 TCGA-BRCA patients with overall-survival data. High expression (red) denotes values above the gene-specific cohort median; low expression (blue) includes values at or below the median. Tick marks indicate censored observations. Time is shown in days. Nominal log-rank P values are 0.053 and 0.070, respectively. Neither comparison met the nominal $P < 0.05$ threshold. Results for all 11 targets are provided in Supplementary Table S6.
