# Supplementary material for: Cell-resolved spatial mapping of antibody-drug conjugate targets in breast cancer
Source: Transl Oncol. 2026 Oct 12;74:103068. doi: 10.1016/j.tranon.2026.103068 (PMC13679307; doi:10.1016/j.tranon.2026.103068)
Supplement: Supplementary file 2 [file mmc2.pdf]

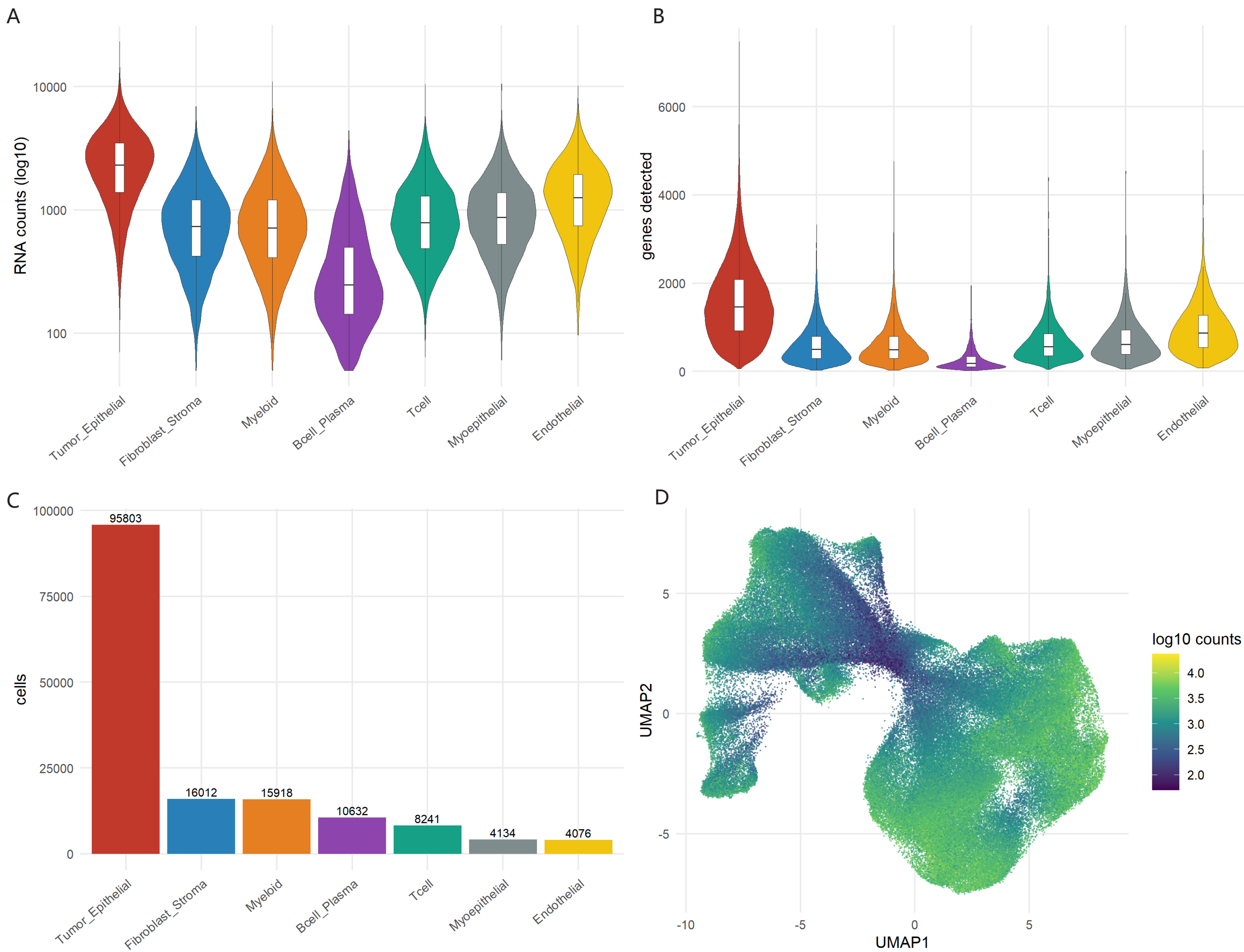

Figure S2. Quality control of the single-cell data. All panels summarize the 154,816 analyzed cells from the CosMx discovery specimen. (A) Total RNA counts per cell by cell type, with a log10-scaled y-axis. (B) Number of detected genes per cell by cell type. In A and B, violin widths show the distributions and internal boxes summarize the median and interquartile range. (C) Cell counts for the seven annotated cell types. (D) UMAP colored by log10 total RNA counts per cell. The reported median cell had 1,531 counts and 999 detected genes.
