# Supplementary material for: Cell-resolved spatial mapping of antibody-drug conjugate targets in breast cancer
Source: Transl Oncol. 2026 Oct 12;74:103068. doi: 10.1016/j.tranon.2026.103068 (PMC13679307; doi:10.1016/j.tranon.2026.103068)
Supplement: Supplementary file 3 [file mmc3.pdf]

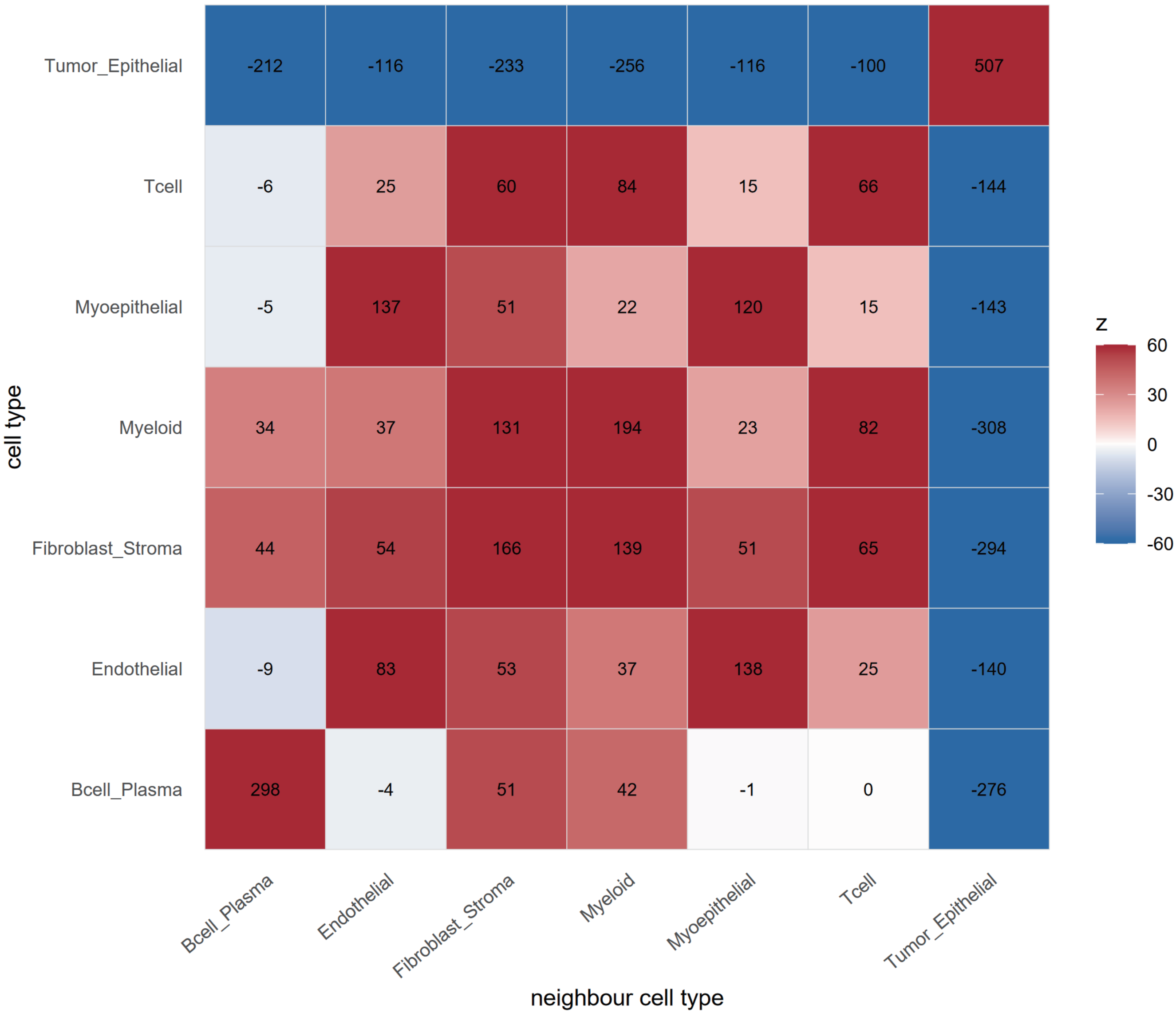

Figure S3. Spatial neighborhood enrichment between cell types. The heat map shows permutation z-scores for ordered cell-type adjacency on the cell-centroid k-nearest-neighbor graph (k = 10; 100 cell-label permutations). Rows identify the index cell type and columns identify its neighbors. Because adjacency is ordered, the matrix is not required to be symmetric. Positive values indicate more adjacency than expected under label permutation; negative values indicate less adjacency. Colors are capped at z = -60 and z = 60, while the labels show uncapped z-scores rounded to integers. All cells come from the single CosMx discovery specimen.
