# Supplementary material for: Cell-resolved spatial mapping of antibody-drug conjugate targets in breast cancer
Source: Transl Oncol. 2026 Oct 12;74:103068. doi: 10.1016/j.tranon.2026.103068 (PMC13679307; doi:10.1016/j.tranon.2026.103068)
Supplement: Supplementary file 4 [file mmc4.pdf]

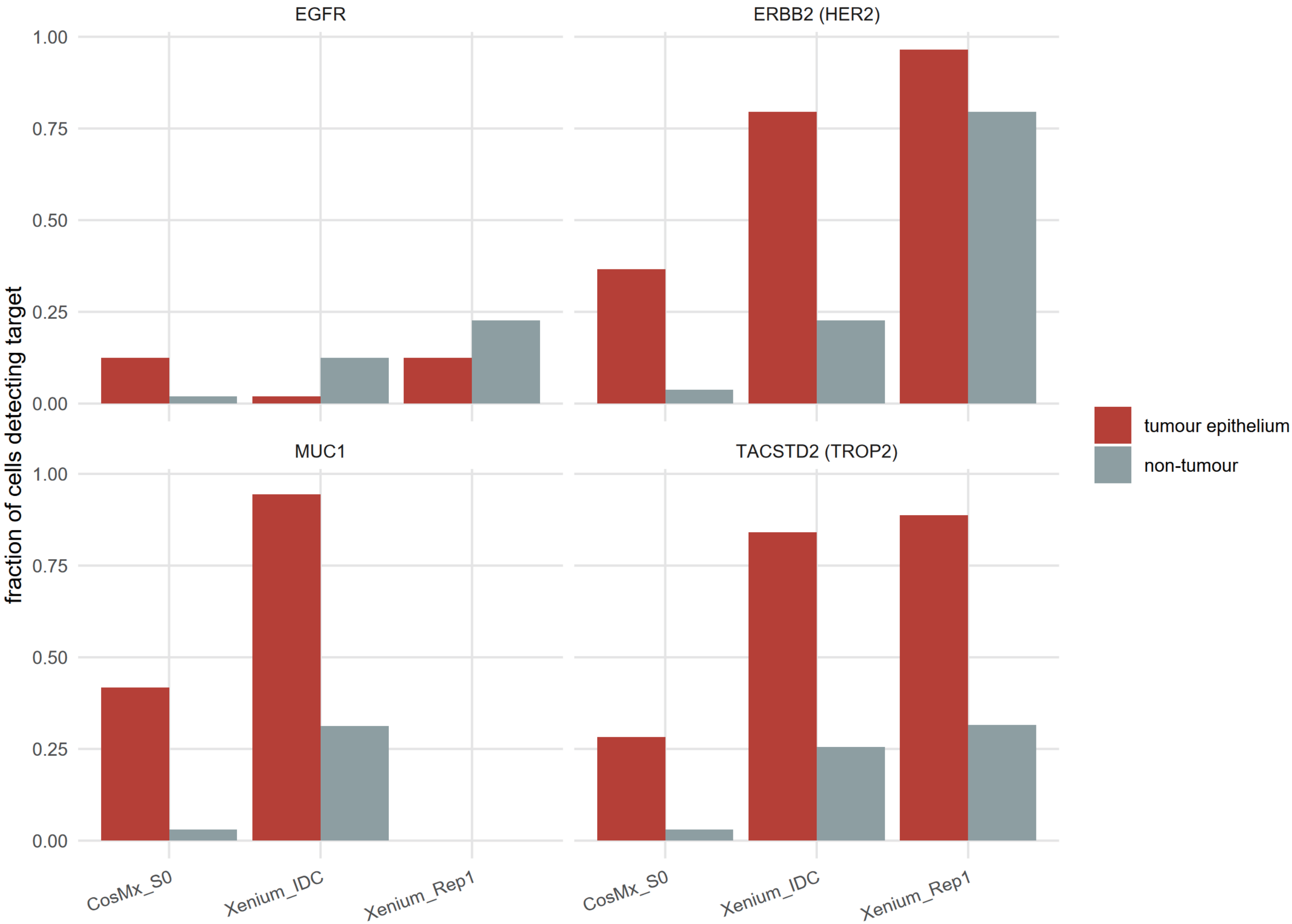

Figure S4. Cross-specimen assessment of ADC-target tumor enrichment. Detection summaries for tumor epithelium (red) and non-tumor compartments (gray) in the CosMx discovery specimen and two Xenium specimens. For CosMx_S0, the non-tumor value is the unweighted mean of detection fractions across six non-tumor lineages. For Xenium_IDC and Xenium_Rep1, it is the pooled fraction of all non-tumor cells with detectable expression. Thus, the non-tumor summaries are not identically weighted across platforms. Xenium_IDC is the invasive ductal carcinoma specimen from a different patient; Xenium_Rep1 is the reference block, whose patient-level independence was not established by public metadata. The missing MUC1 bars for Xenium_Rep1 indicate that no measurement is provided, not zero expression. HER2 and TROP2 detection is higher in tumor epithelium in every specimen. The source values and definitions are given in Supplementary Table S3.
